# Treatment Outcome of Acute Intussusception in Children Under Two Years of Age: A Prospective Cohort Study

**DOI:** 10.7759/cureus.7729

**Published:** 2020-04-18

**Authors:** Nguyen Thanh Xuan, Nguyen Huu Son, Ho Huu Thien

**Affiliations:** 1 Department of Abdominal Emergency and Pediatric Surgery, Hue Central Hospital, Hue, VNM; 2 Pediatrics, Hue Central Hospital, Hue, VNM

**Keywords:** intussusception, pneumatic reduction, surgical intervention

## Abstract

Background

Intussusception is a common cause of small intestinal obstruction in children under two years of age. Late diagnosis can lead to a potentially worse condition. This prospective study aims to describe the clinical manifestation and develop a conservative management protocol for acute ileocaecal intussusception in children under two years of age.

Methods

This prospective study was carried out in 118 consecutive patients under two years of age. Patients presented with symptoms and signs of acute intestinal obstruction and a diagnosis of ileocaecal intussusception confirmed by ultrasound were included in this study. All the patients were managed with either pneumatic reduction or operation.

Results

There were 70 boys and 48 girls ranging in age from three months to two years with a median of 12.5 months. Clinical presentation included abdominal pain (100%), vomiting (82.2%), bloody stool (11.9%), and a palpable mass (43.2%). Patients hospitalized with the symptoms and signs for less than 24 hours accounted for 80.5% of the cases. The overall success rate of pneumatic reduction was 98.3%. Late hospital admission (≥ 24 hours from illness onset), bloody stool, and presenting with the classic triad of symptoms of intussusception were found as the factors that correlated to the surgical management outcome. All patients recovered well without any complications. The median of postoperative hospital stay of two days for the pneumatic reduction group and six days for the operation group.

Conclusion

The early diagnosis of intussusception contributes to the success of pneumatic reduction and reduces the requirement of surgical intervention.

## Introduction

Intussusception is the most common cause of small intestinal obstruction in children under two years of age [1-2]. It happens when one segment of the small bowel invades or telescopes in the distal bowel, leading to bowel wall edema and venous congestion. There are approximately 74 intussusceptions per 100,000 infants occurring annually worldwide [3]. The incidence has been reported as high in Vietnam and Korea with 300 per 100,000 infants and as low in Bangladesh with nine per 100,000 infants [3].

Usually, acute intussusception is presented with vomiting, colicky abdominal pain, and bloody stool if late admission [4]. To approach the final diagnosis, some imaging tests can be performed such as ultrasonography, abdomen X-ray, barium study, and computed tomography in less certain cases [5-6].

Some infants with intussusception can recover spontaneously [7-9]. Persistent small intestinal intussusception, however, can cause a lead point or bowel necrosis and would require surgical intervention. If acute intussusception is not recognized and treated timely, the arterial blood supply to the bowel may be blocked, causing bowel infarction and perforation. Untreated intussusception is a potentially worse condition or even fatal outcome [7-9]. Therefore, physicians should recognize such cases as soon as possible and indicate the correct management.

Recently, the patients are often hospitalized in an early stage with minimal symptoms, including abdominal pain and vomiting. Thus, the confirmed diagnosis is mainly based on ultrasound findings, and a pediatric surgeon should decide whether to take the infant for an enema pneumatic reduction or close surveillance [10-11]. Besides, choosing an intervention between saline-enema and pneumatic reduction for the treatment of acute intussusception is under debate due to the varied success rate of 83%-88% [12-15]. This prospective study aims to describe the clinical manifestation and develop a conservative management protocol for acute ileocaecal intussusception in children under two years of age.

## Materials and methods

Patients

This prospective study was carried out on 118 consecutive patients under two years of age, at the Pediatric Center of Hue Central Hospital (Hue City, Vietnam), from May 2017 to May 2019 after the ethical approval of the Research Ethics Committee under reference number 01012017/HCH. Written consent was obtained from all patient’s parents.

Patients presented with symptoms and signs of acute intestinal obstruction and a diagnosis of ileocaecal intussusception confirmed by ultrasound were included in this study. Patients with spontaneously conducted intussusception and those who had another diagnosis were excluded from the study.

**Figure 1:**
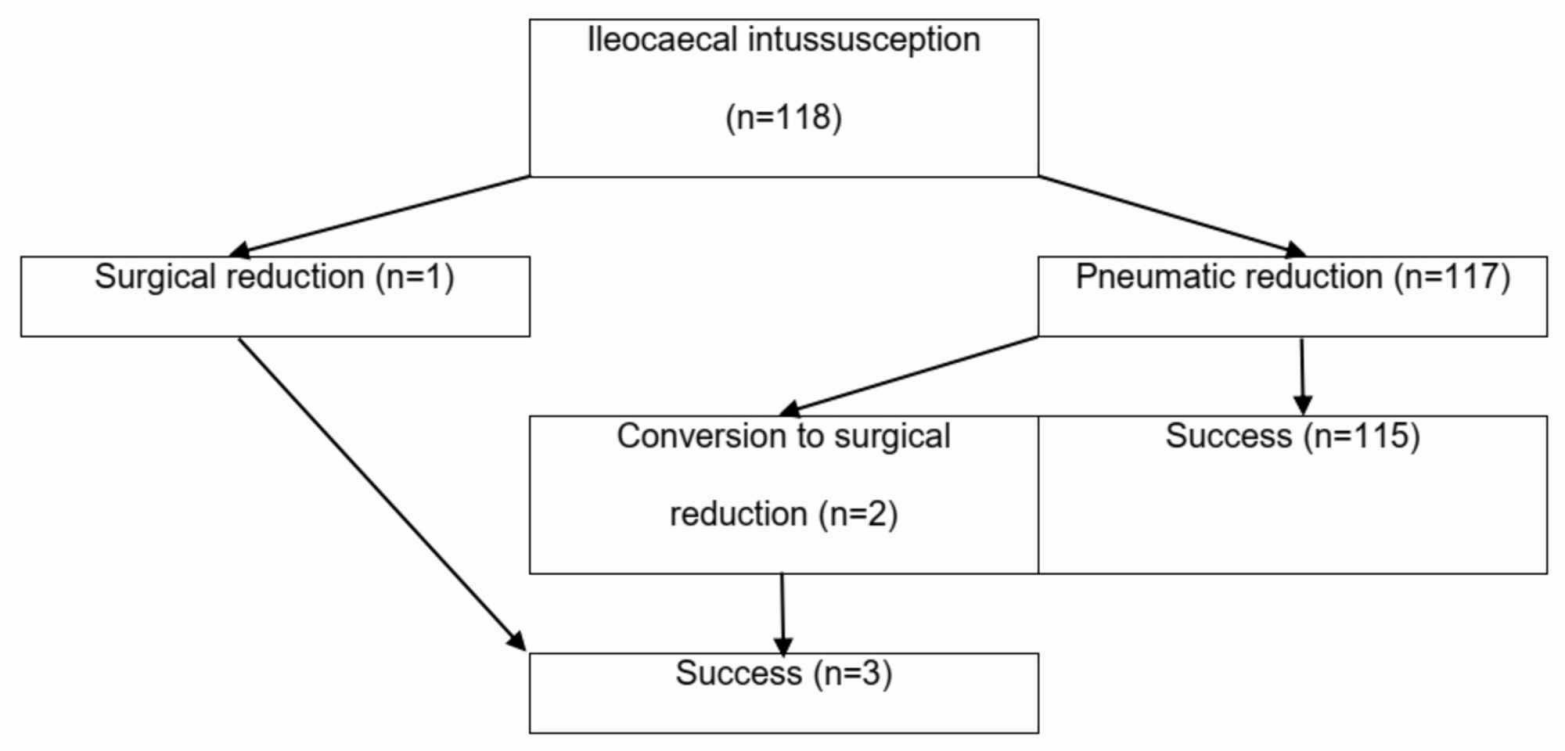
Patient flowchart

Other investigations, such as X-ray, water-soluble contrast study, and computed tomography of the abdomen, were performed as per the requirements of specific patients. Total blood count, serum electrolyte, and urea-creatinin levels were done. The patients were immediately taken for either pneumatic reduction or operation due to the intussusception (Figure 1).

Treatment protocol

Pneumatic Reduction

Once intussusception is diagnosed, the next step is to attempt using pneumatic reduction under mask anesthesia.

**Figure 2:**
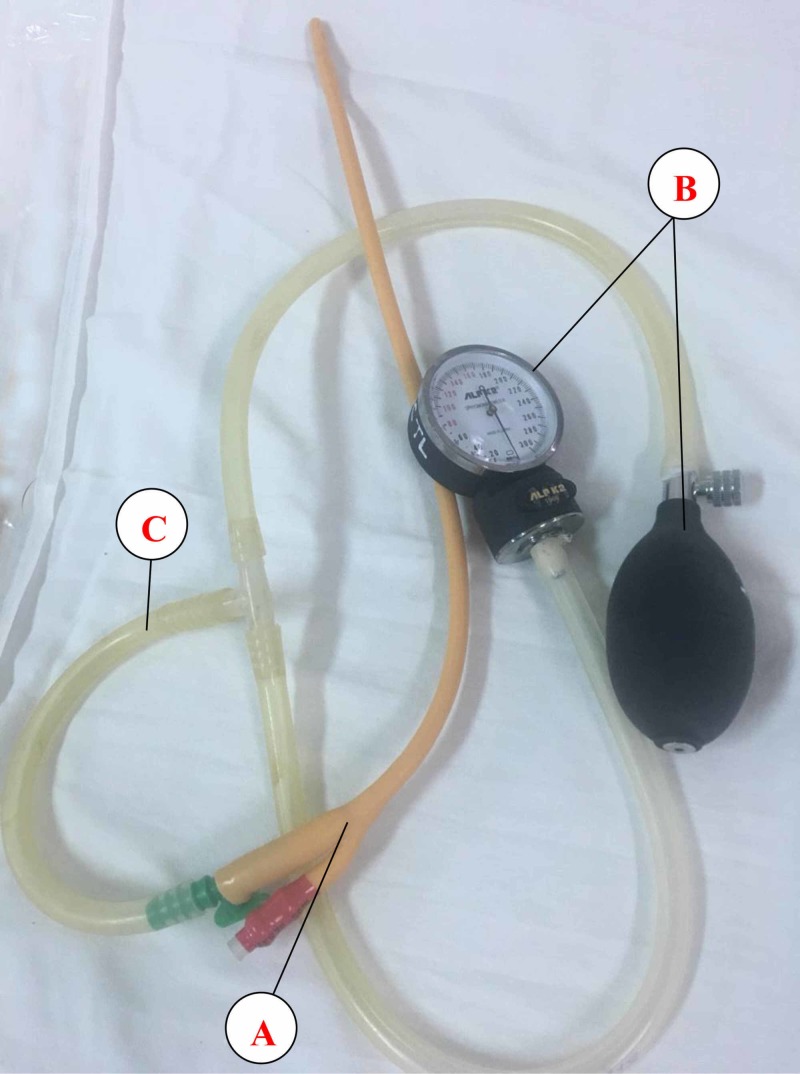
The device was constructed from a Foley catheter (Procath, Ishwari Healthcare Pvt. Ltd.) (A), an ALPK2 sphygmomanometer (Japan) (B), and plastic tubes of an appropriate size (C). It was completely sterilized before use.

A nasogastric tube was positioned and soaked in a water tray. A specific device was assembled with available equipment in the hospital. A handheld pump attached to a pressure gauge, a two‑way Foley’s balloon catheter (20 Fr) was used to insufflate the air through the rectum (Figure 2). After the catheter had been introduced per rectum, the balloon was inflated with between 20 and 40 ccs of water. It completely occluded the rectum by pulling the catheter while the patients’ gluteal folds were strapped together by an assistant during the procedure. The air was handly insufflated with a pressure of between 100 and 120 mmHg. The pressure was maintained for three minutes. After one minute, the insufflation was repeated. A maximum of three insufflations was performed. Reduction of the intussusception was identified during the procedure by signs of the air coming out of the nasogastric tube and the abdomen becoming equally round. The patient’s vital signs were monitored throughout the procedure with the monitor. A flat abdominal X-ray was performed for the patients to ensure complete reduction. If there was any suspicion of the intussusception remaining, abdominal ultrasound was taken for exploration two hours after the procedure.

Surgical Intervention

If the intussusception did not reduce after the three attempts, the pneumatic reduction was considered a failure and the patient proceeded to surgery.

Endo-tracheal anesthesia was used instead of mask anesthesia for all cases. The abdomen and bowel are typically explored through a transverse incision in the right lower quadrant (RLQ) or a right transverse supraumbilical incision dependent on the intussuception mass. After inspection for signs of perforation, the intussusception is identified and delivered into the wound. First, an attempt is made at a manual reduction by retrograde milking of the intussusceptum. Although gentle pulling may aid in reduction, vigorous pulling apart of the intussuscepted segment of the bowel must be avoided.

After a successful manual reduction, the involved bowel segment may appear edematous, hyperemic, or ischemic, but such findings do not necessarily mandate resection. An appendectomy is always done to prevent intussusception recurrence.

If the manual reduction is unsuccessful, if a mass or lead point is present, or if perforation has occurred, segmental bowel resection is necessary. After resection, a primary anastomosis may be performed.

Data collection

Data were collected using the medical records of the pediatric examination room, operating room, post-surgery, intensive care, and patients' records. The data concerned demographics, delay of admission, clinical symptoms, surgical procedures, and duration of hospitalization.

All the data were analyzed statistically expressing data values as percentages, mean, standard deviation, or median values as per the data type (qualitative or quantitative). The chi-squared test was used for qualitative data. p<0.05 was taken as significant.

## Results

A total of 118 pediatric patients admitted with acute intussusception were included in the study. Acute intussusception was seen to affect more males (70 patients, 59.3%) than females (48 patients, 40.7%). The most common occurrence was in the age group of 13-24 months (72 patients, 61.0%), followed by the group aged five to 12 months (44 patients, 37.3%), while the least common occurrence was in the age group ≤ four months (two patients, 1.7%). The median age of our patients was 12.5 months (IQR: 7.0 - 20.3 month). Most of the patients (80.5%) were hospitalized before 24 hours from illness onset, while there were nine cases (7.6%) admitted too late (Table 1).

**Table 1:** Patient’s characteristics IQR: interquartile range

Characteristics	All patients (n=118)
Sex, n(%)	
Male	70 (59.3)
Female	48 (40.7)
Age group, n(%)	
≤ 4 months	2 (1.7)
5 – 12 months	44 (37.3)
13 – 24 months	72 (61.0)
Age (median, IQR) (month)	12.5 (7.0 – 20.3)
Time from illness onset to hospital admission, n(%)	
< 24 hours	95 (80.5)
24 – < 48 hours	14 (11.9)
≥ 48 hours	9 (7.6)
Abdominal ultrasound performed, n(%)	118 (100)
Flat abdominal X-ray performed, n(%)	20 (16.9)
Computed tomography performed, n(%)	1 (0.8)

In addition, to confirm intussusception and detect complications, ultrasound, flat abdominal X-ray and computed tomography were performed to 118 (100%), 20 (16.9) and 1 (0.8%) patients, respectively (Table 1).

Table 2 shows the clinical presentation of children with acute intussusception. The classical presentation of abdominal pain (excessive crying), vomiting, constipation, and abdominal distension was observed in 118 (100%), 97 (82.2%), 41 (34.7%), and 27 (22.9%), respectively. Our study had only 14 patients with bloody stools, accounting for 11.9%. The signs of a palpable mass were found in 51 (43.2%) of patients upon physical examination.

**Table 2:** Clinical features

Clinical Features	Number of Patients	%
Abdominal Pain	118	100
Vomiting	97	82.2
Constipation	41	34.7
Abdominal distension	27	22.9
Fever	10	8.5
Bloody stool	14	11.9
Palpable mass	51	43.2
Diarrhea	8	6.8
Classic triad of symptoms of intussusception^*^	14	11.9

Among 118 patients, only one patient was initially treated by surgical reduction and 117 were managed by pneumatic reduction. Out of the pneumatic reduction cases, two patients were transferred to operation due to failed intervention. All three cases undergoing an operation were successfully manually reducted of the intussusception and no additional surgical management was required.

All 118 patients were well-recovered without any complication. They were discharged with a median postoperative hospital stay of two days for the nonsurgical group and six days for the operation group.

Late hospital admission (≥ 24 hours from illness onset), bloody stool, and presenting with the classic triad of symptoms of intussusception were found as the factors that correlated to the surgical management outcome (Table 3).

**Table 3:** Related fators of surgical management

Characteristics	Surgical group (n=3)	Non-surgical group (n=115)	p-value
Age			
≤ 12 months	2	22	0.0870
> 12 months	1	93	
Time from illness onset to hospital admission			
< 24 hours	0	98	0.0166
≥ 24 hours	3	17	
Bloody stool			
Yes	3	11	0.0071
No	0	104	
Duration of bloody stool			
< 5 hours	1	1	0.3182
≥ 5 hours	2	10	
Presenting with the classic triad of symptoms of intussusception			
Yes	2	12	0.0243
No	1	103	

## Discussion

Intussusception in infants is a surgical emergency that requires rapid diagnosis and timely intervention. It happens when a segment of the bowel (the intussusceptum) invaginates into the lumen of another segment of the bowel (the intussuscipiens). Both the small and large intestine can be affected but the most common type of acute intussusception occurs at the junction between the cecum and the ileum and is called the ileocecal intussusception [16].

In our results, the median age of the patients was 12.5 months - the highest incidence of intussusception was in the age group of 13-24 months. Almost previous studies showed that acute intussusception was common in children under two years of age [17]. However, the results in the study of Giak and Satter noted the most common age group was under one year old [18-19].

Our study showed that intussusception occurred in males more than in females with a male:female ratio of 1.5:1. These results were consistent with other reports. A remarkable increase in the number of male patients with intussusception as compared to female patients has been reported in lots of previous studies [20]. Consequently, many studies have used the condition of a boy <1 year of age as a secondary criterion for intussusception diagnosis [21].

We demonstrated that children under two years of age with intussusception presented with a wide range of clinical symptoms, which highlights the importance of describing them as carefully as possible in medical records to facilitate further evaluation of the diagnoses. Most previous studies have reported that the classic triad of symptoms (vomiting, abdominal pain, and rectal bleeding or bloody stools) were present in 10%-66% of cases [22]. In our study, the triad was present in 11.9% of the cases while the most frequent symptom (100%) was abdominal pain, which was consistent with other studies [23-24]. Abdominal pain had a high positive predictive value of 99%, a high negative predictive value of 100%, and a high sensitivity 90% but a specificity of just 19% when validated within the Brighton criteria for intussusception [24].

Regarding other clinical features, the rate of a palpable mass was 43.2% in our study, which was lower than that in the study of Saez-Llorens with 51% [25]. According to Marsicovetere, in children with intussusception, the physical exam may reveal a palpable “sausage-shaped mass” in the right upper quadrant or epigastric region of the abdomen, but the mass is only detected in approximately 60% of cases [26].

Most patients were often hospitalized at an early stage so that signs of bloody stool were presented in small patients that accounted for a lower percentage than that in the study of Saez-Llorens (48%) and Schollin Ask (38%) [22,25].

Patients presenting with vomiting in our study were accounted for 82.2%, which was not different from other studies. Vomiting is the most common symptom of intussusception, especially in infants under four months of age. Initially, the vomit contains only food from the stomach, but in the late stage when the bowel is completely obstructed, bile-stained vomiting may be seen.

The success rate of the pneumatic reduction in our study was very high. This was the result of an appropriate diagnosis and choosing the right treatment method, in terms of satisfying the indications and contraindications of pneumatic reduction in order to avoid surgical management. Another reason was that our surgical team performed the procedures in the operating room with the patients initially anesthetized and a nasogastric tube inserted, using an air pump with a blood pressure monitor to check the pressure, thus avoiding complications during the procedure such as regurgitation or colon perforation. Neither ultrasound exploration nor monitoring under the fluorescent screen was performed to check the intussusception reduction during the procedure because ultrasound is interfered with by air and X-ray may cause exposure for the patients.

In our study, the group of patients treated with the non-surgical method had the shortest duration of hospital stay, with a median of two days. The patients were discharged from the hospital after one to two days if there were no complications or unusual signs. The short hospital stay is one of the advantages of the non-surgical method, which is very economically and psychologically beneficial for patients, as well as for avoiding hospital infections.

Many studies have been conducted to investigate the clinical and ultrasound factors that help predict the tightness of the intestinal obstruction, thereby allowing for appropriate measures to be taken to avoid prolonging the disease, reducing the complications of the disease, and increasing effectiveness when doing non-surgical procedures to reduce the intussusception [27].

In order to successfully treat intussusception by the pneumatic reduction method, there should be two factors: (1) Early detection of intussusception, indicating the necessary treatment from the first symptoms; (2) Upon a patient’s admission, all symptoms need to be detected in order to know the prognostic factors of the condition of the intestinal obstruction so as to get the proper indication-pneumatic reduction or operation.

According to Katz el at., a duration of intussusception of less than 12 hours, no bloody stool, no bowel obstruction, no palpable masses, and no dehydration are prognostic factors for the success of the pneumatic reduction method (p <0.001) [28].

A relationship between hospital admission time and treatment therapeutics was noted in our study. This result was consistent with the study of Reijnen [27]. Other studies have concluded that the duration of intussusception is related to the postoperative outcome - the longer the duration of intussusception, the lower the success rate of pneumatic reduction and the greater the surgery rate. In summary, upon first contact with the patient, pediatricians should detect the disease as soon as possible from the first symptoms presenting at the onset. They should not rule out acute intussusception in favor of the diagnosis of a different medical condition. As a result, the treatment will become easier and more effective, avoiding unnecessary complications and surgical treatment.

Many previous studies have shown a relationship between the duration of the appearance of the bloody stool and surgical outcomes [27]. According to Kuppermann, bloody stools were one of the most important prognostic factors [29]. In our study, the higher the percentage of patients with bloody stools, the higher the surgery rate. If a patient presented with the classic triad of symptoms of intussusception, the likelihood of surgery increased. Thus, we should first think of acute intussusception if an infant presenting with at least one of these symptoms: vomiting, colicky abdominal pain, and bloody stool, and then perform an abdominal ultrasound to confirm or rule out the diagnosis.

Therefore, Brighton's criteria for intussusception should be applied in the early diagnosis and timely treatment of patients in order to reduce the rate of complications and deaths due to this disease.

## Conclusions

This study provides important data on baseline clinical characteristics and a very high success rate using a pneumatic reduction method for the treatment of acute intussusception in children under two years of age. A delay in presentation results in a decrease in the success rate of pneumatic reduction and increases the risk of surgical intervention.
